# Antero-lateral transthoracic endoscopic approach for a calcified thoracic disc herniation

**DOI:** 10.3171/2022.3.FOCVID221

**Published:** 2022-07-01

**Authors:** Roque Carlos Fernández, Miguel Mesa, Daniel Rosenthal, Victor Rodrigo Paradells

**Affiliations:** 1 Department of Neurosurgery, Hochtaunus Kliniken, Bad Homburg vor der Höhe, Germany;; 2 Department of Thoracic Surgery, Clinica Universidad de Navarra, Pamplona, Spain; and; 3 Department of Neurosurgery, Clinica Universidad de Navarra, Pamplona, Spain

**Keywords:** calcified, endoscopic approach, minimally invasive, spine surgery, thoracic disc herniation, transthoracic

## Abstract

Thoracic disc herniation is one of the most therapeutically challenging spine conditions. A myriad of surgical approaches have been described in the literature, including posterior, anterior, and combined techniques. However, transthoracic and retropleural approaches are currently deemed the most effective techniques to successfully obtain anterior decompression. Herein the authors describe a 65-year-old female patient who underwent a transthoracic endoscopic approach to remove a calcified herniated thoracic disc that caused spinal cord compression. Despite having a long learning curve, the surgical technique described herein can be even used in patients with complex and calcified thoracic disc herniations.

The video can be found here: https://stream.cadmore.media/r10.3171/2022.3.FOCVID221

## Transcript

This is a video to present an anterolateral transthoracic endoscopic approach for a calcified thoracic disc herniation (TDH).

### 0:29 Case Description.

This is a 65-year-old woman with a past medical history of asthma, right nephrectomy, and pulmonary embolism (PE). She was diagnosed of a large, calcified T5–6 TDH, after a chest CT done as a follow-up for her pulmonary embolism. The neurological examination was within normal limits, without motor or sensitive symptoms. On further questionnaire, she described a mild urinary incontinence that she has had for years, and she explained it because of her age and births.

### 1:03 Preoperative Images.

This is a sagittal and axial CT without contrast. It shows a calcified TDH at T5–6 with significant stenosis of the spinal canal. We notice the calcification of the thoracic hernias as a common finding. The axial view shows a wide bone implantation.

### 1:21 Surgical Plan and Risks.

The T2-weighted images MRI shows that a deformed/displaced spinal cord with probable signs of myelopathy. This compression needs to be addressed from an anterior approach to achieve a complete decompression reducing the risk of spinal cord injury that a posterior approach could carry. Despite the fact that the compression is mainly the left side, we planned the approach from the right. A simple decompression could have been achieved from the left side, but a correct stabilization with screws is very dangerous at this level due to the aorta. This is the reason why we approached the hernia with subsequent osteotomy from the right side. It is important to make sure the osteotomy is long enough to achieve a sufficient decompression.

### 2:12 Positioning and Patient Preparation.

The patient is right lateral position. The surgeon is in her ventral side. We do not usually use intraoperative neuromonitoring, as we need sufficient relaxation of the diaphragm, just in case it had to be retracted. A 30° endoscope is used. Given the size and depth of the thoracic cavity, long spine surgical instruments were also utilized. Usually, we place intramuscular needles to count vertebras with x-ray. We also use a lumbar puncture needle to localize the exact intrathoracic space, leaving it inside as a reference to find with the endoscope. We approach the patient using portals at the middle and anterior axillary lines.

### 3:01 Surgical Procedure.

Localizing of needle: The first portal is for the camera. We try to find the needle we placed previously to localize the correct rib. We follow it medially to reach the exact intervertebral space. You can also count the ribs to ensure the level.

Visualizing other portals: With this first portal we can see from inside how we open the others.

Ending the lung collapse with retractors: We use a lung retractor to make our field more comfortable.

Subperiosteal dissection: Once we reach the rib, subperiosteal dissection is performed. In this case, the corresponding rib we aim is the sixth.

Head of the rib osteotomy: Next step is to remove a piece of bone from the head of the rib, which we will use as autologous bone graft to achieve the fusion posteriorly.

Then, we direct our attention to the main field and start to make small thin osteotomies with the bone scalpel. This is a very useful instrument for this kind of surgery, because it can make thin slices of the bone and help us to decompress securely from the side of the bone. We use the bone scalpel, osteotomes, and rongeurs. It is important to make small bone osteotomies in order to minimize the likelihood of neural damage. The bone scalpel allows making straight cuts, specifically in the bone, with a very low risk of harming the spinal cord and/or other soft tissues.

Dural exposure: We finally arrive to the epidural space and make it wider following the dura.

Possible durotomy: Here, during the decompression, we notice a possible durotomy, that we are capable to seal it temporarily with Surgicel and a cottonoid patty while we keep opening the space with rongeurs.

Last bone fragment: We are removing the last bone fragment that is compressing the spinal cord to achieve finally a complete decompression. And we confirm it with a hook.

Sealing possible durotomy: To prevent a CSF leak due to the questionable durotomy, we use TachoSil, Spongostan, and Surgicel.

Bone graft (head of the rib): We place the bone graft in the intervertebral space (head of the rib).

Tapping and screws: Then, we use the bone scalpel to make the initial hole for the screws and tapping, and finally we put the screws.

Rod: The rod is placed carefully to avoid its loss in the thoracic space. If that happens, it can become an issue as it would be very difficult to find it. The nuts are positioned and adequately torqued.

Topic glucose: Topic glucose solution is used to create a controlled and localized pleuritis and increase the sealing of the leak.

Drain tube: We insert the drain tube under the direct endoscope vision, keeping in mind not placing it too cranial, as it could cause more pain and cough during postoperative course.

### 8:06 Surgical Outcome.

The postoperative CT scan revealed a complete resection of the hernia and a good decompression. We confirmed that the osteotomy was accurate and sufficient to decompress the spinal cord, as we planned at the beginning. The fragment of the rib that we used as bone graft was correctly placed to achieve fusion. The patient woke with paresthesia in the left leg as a new symptom that almost completely disappeared in the next month. The chest tube was removed in the 2nd day. We usually remove it in the 1st day, but the use of topic glucose can carry a subsequent pleuritis that can lead to a significant pleural effusion—reason why we kept it for an additional 1 more day. The patient was discharged on the 5th postoperative day. In the next month of the surgery, she also noticed an improvement of her urinary continence.

### 8:54 References^1–5^

## Disclosures

The authors report no conflict of interest concerning the materials or methods used in this study or the findings specified in this publication.

## Author Contributions

Primary surgeon: Rodrigo Paradells, Fernández. Assistant surgeon: Rodrigo Paradells, Mesa. Editing and drafting the video and abstract: Rodrigo Paradells, Mesa, Rosenthal. Critically revising the work: Rodrigo Paradells, Rosenthal. Reviewed submitted version of the work: Mesa. Approved the final version of the work on behalf of all authors: Rodrigo Paradells. Supervision: Rosenthal.

## Supplemental Information

### Patient Informed Consent

The necessary patient informed consent was obtained in this study.
